# Retrospective evaluation of attempted vaginal deliveries in dichorionic twin pregnancies

**DOI:** 10.1007/s00404-020-05882-y

**Published:** 2020-11-21

**Authors:** Sabine Enengl, Peter Oppelt, Simon-Hermann Enzelsberger, Philip Sebastian Trautner, Omar Shebl, Birgit Brandl, Richard Bernhard Mayer

**Affiliations:** 1 Department of Gynecology, Obstetrics and Gynecological Endocrinology, Kepler University Hospital, Johannes Kepler University Linz, Altenbergerstrasse 69, 4040 Linz, Austria; 2 Department of General Surgery, Pyhrn-Eisenwurzen Klinikum Steyr, Steyr, Austria; 3 Department of Gynecology and Obstetrics, Konventhospital Barmherzige Brueder Linz, Linz, Austria

**Keywords:** Multiple pregnancies, Twin delivery, Birth mode, Preterm birth

## Abstract

**Purpose:**

Numbers of planned cesarean deliveries are increasing in twin pregnancies, despite the lack of evidence for this approach, and the second twin is thought to be at risk for a poorer outcome. The aim of this study was to examine whether twins have a poorer outcome if an attempted vaginal delivery is changed to a cesarean section or combined delivery.

**Methods:**

This retrospective data analysis included all women with dichorionic twin pregnancies attempting vaginal delivery over a 10-year period. Outcome parameters for the first and second twins relative to their mode of birth were compared. A correlation model between the interdelivery time interval and Apgar scores was calculated. Subgroup analyses assessing the birth mode of the first and second twins were conducted.

**Results:**

A total of 248 women were enrolled in the study. The second twins had significantly lower values for outcome parameters, such as umbilical artery cord pH and Apgar scores in comparison with the first twins (*P* < 0.01). The subgroup analysis of birth modes in first and second twins showed a significantly poorer outcome in the cesarean section and combined delivery group (*P* < 0.05). The interdelivery time interval was significantly longer in the second twin cesarean section group (*P* < 0.01). There was no significant correlation between the interdelivery time intervals and Apgar scores (*P* > 0.05).

**Conclusion:**

Although outcome parameters were significantly lower in second twins and twins born via secondary cesarean section, the clinical relevance of this appears to be negligible.

## Introduction

Numbers of twin pregnancies are still increasing due to artificial reproductive technologies [1–3], and they account for approximately 1–3% of the total [4]. Twin deliveries are associated with higher rates of perinatal morbidity and mortality due to prematurity, intrauterine growth restriction, and complications resulting from monochorionicity [5–9]. The second twin appears to be at greater risk than the first twin [10]. A persistent increase in the planned cesarean sections is therefore being seen in Europe, despite the lack of evidence for this approach and women’s preference for vaginal birth [11–14].

Several studies have been conducted to investigate the impact of various factors on the neonatal outcome for both twins, with contradictory results. A large randomized controlled trial, the Twin Birth Study, concluded that a planned vaginal delivery does not significantly influence neonatal and maternal mortality and morbidity in comparison with a planned cesarean section if the first twin is in cephalic presentation, both twins are of normal size, and the second twin is not significantly larger than the first [15]. The study also reported that the second twin was more likely to have neonatal complications than the first, although planned cesarean section did not reduce this risk [15]. Other studies have shown that maternal risk factors, such as peripartum hemorrhage, are more likely to occur in surgical twin deliveries [16]. Postoperative maternal infections are more often reported in unplanned cesarean section deliveries, but these are rare [16].

As the second twin is more likely to have a poorer outcome due to complications, such as cord prolapse, fetal distress, and the difficulty of intrapartum monitoring, potential risk factors have been investigated [10, 17]. Some studies have reported lower Apgar scores and umbilical artery pH in second twins after vaginal or combined delivery in comparison with planned cesarean section [16–18]—but again with a low prevalence of these events.

There is as yet no consensus regarding an optimal interdelivery time interval. Some authors have recommended an upper time limit of 30 min, reporting a significant impact on the outcome for the second twin when this limit is exceeded [5, 14, 19]. A longer interdelivery time interval is also said to carry a higher risk of a combined vaginal–surgical delivery [6, 20]. To keep the interdelivery time interval short, active management of the second twin’s delivery would have to be advocated [5]. With dichorionic twins, however, some authors have not observed any significant differences in outcomes in relation to longer interdelivery time intervals [5].

The present study focuses on comparison of the first and second twins in attempted vaginal deliveries. Most of the literature reports have evaluated differences between vaginal birth and planned cesarean delivery [13, 15, 16, 21]. The aim of this retrospective analysis was to determine whether an intrapartum change in the delivery mode from vaginal to cesarean or combined has any influence on the outcome for each of the twins.

## Methods

All women with twin pregnancies intending to attempt vaginal delivery at Kepler University Hospital, Linz, Austria, from 2007 to 2017 were included. A total of 248 twin births and 496 twins were enrolled in the study, on the basis of a retrospective analysis of medical records and the obstetric clinical database. Criteria for a vaginal attempt were cephalic presentation of the first twin, an estimated birth weight of both fetuses > 2400 g, or completion of gestational week 32 and a dichorionic, diamniotic twin pregnancy. The gestational week at the time of delivery was calculated on the basis of crown–rump length using ultrasound during the first trimester. Chorionicity was determined on the basis of the presence or absence of the lambda sign on the first available ultrasound.

Maternal or fetal indications for delivery, as well as intrapartum management, were based on the hospital’s standard operating procedures and on the decision making of the obstetrician in charge. Induction of labor in dichorionic twin pregnancies is recommended at 38 + 0 weeks of gestation at the latest. Epidural anesthesia is not mandatory. After delivery of the first twin, an ultrasound and digital examination is usually carried out. If the second twin is presenting in the transverse position, an attempt at external version is made. If there is a lack of contractions, amniotomy, or oxytocin may be used. The interdelivery time interval was defined and calculated between the delivery of the first and the second twin. The aim of the study was to compare the first and second twins relative to time of delivery/interdelivery interval, mode of delivery, birth presentation, and neonatal outcome parameters such as the Apgar score, umbilical artery pH, birth weight, or transfer to neonatal units. Severe acidosis was defined as an umbilical artery pH below 7.10. Correlations between the interdelivery time interval and the second twin’s Apgar score were analyzed in the subgroup with the first twin born vaginally. Subgroup analyses comparing vaginal or cesarean delivery of the first and second twins were carried out.

### Statistical analyses

All datasets for continuous variables were checked for normal distribution (Kolmogorov–Smirnov test with Lilliefors significance correction, type I error 10%). Twin comparisons of continuous variables with normally distributed datasets were carried out using the paired *t* test; otherwise, the Wilcoxon test was used. Nominal variables were compared using the McNemar test and the exact one-sample chi-square test. Subgroup comparisons of continuous variables with normally distributed datasets were conducted using the *t* test for independent samples (test for variance homogeneity: Levene test, type I error 5%); otherwise, the Mann–Whitney *U* test was used. Nominal variables were compared using Fisher’s exact test or the exact chi-square test.

Correlations (Apgar vs. time from first to second birth) were reviewed using Spearman’s rank correlation coefficients. Two-sided 95% confidence intervals were calculated according to the nature of the data (parametric, nonparametric, or Clopper–Pearson). Type I error was not adjusted for multiple testing. The results of inferential statistics are therefore only descriptive. Statistical analysis was performed using the open-source R statistical software package, version 3.4.1 (R Foundation for Statistical Computing, Vienna, Austria).

### Ethical approval

The study was approved by the local ethics committee of Upper Austria (Ethikkommission des Landes Oberoesterreich/JKU-Ethikkommission) (K-131-17) on April 19, 2017.

## Results

The study included 248 women and 496 twins in dichorionic twin pregnancies in which vaginal delivery was attempted. During the study period, 847 twin births took place at Kepler University Hospital, with 315 primary cesarean sections performed (37.2%). In the patient collective, the number of planned cesarean sections in twin pregnancies remained steady with a minimum of 30% in 2013 and a maximum of 47.6% in 2007.

The median gestational age in the pregnancies analyzed was 36 weeks with a minimum of 34 and a maximum of 41 weeks. 57.7% were preterm births.

The baseline maternal characteristics are shown in Table 1. Maternal risk factors carrying a high chance of cesarean section have been investigated. There was no significant correlation concerning maternal age, body mass index (BMI), or the presence of (gestational) diabetes. Patients with cesarean section of either of the twins showed a significantly shorter duration of pregnancy (first twin 254 days ± 12.89 vs. 261 days ± 11.93; second twin 255 days ± 12.47 vs. 261 days ± 12.31; *P* < 0.01). Indications for induction of labor were preterm (premature) rupture of membranes (*n* = 10) and gestational age (*n* = 18).

**Table 1:** Baseline maternal characteristics

	Mean/median	SD/range
Age (years)	31.6	5.24
Gravidity	2	1–12
Parity	1	1–6
BMI (kg/m^2^)	29.3	8.21
Duration of pregnancy (days)	257.93	12.72

	*n*	%
Gestational diabetes	12	4.8
Induction of labor	28	11.3
Epidural anesthesia	82	33.1
Spinal anesthesia	56	22.6
Mode of delivery		
Vaginal	114	46.0
Cesarean section	108	43.5
Combined	26	10.5

*BMI* body mass index, *SD* standard deviation

There were 114 vaginal deliveries (46.0%), 108 cesarean sections for both twins (43.5%), and 26 combined deliveries (10.5%; vaginal delivery of the first twin and cesarean delivery of the second). The indications for cesarean sections are shown in Table 2. Both twins had cephalic presentations in 171 of the 248 deliveries (69.0%). In 54 cases (21.8%), the first twin was in cephalic presentation and the second in breech presentation. In 23 births (9.2%), the first twin had a cephalic presentation and the second a transverse presentation. In 140 cases (56.5%), the first twin was delivered vaginally, with 123 spontaneous deliveries (87.9%), and 17 assisted (vacuum extraction) deliveries (12.1%). In all, 114 of the second twins were born via vaginal delivery, with 17 spontaneous vaginal breech deliveries (14.9%) and 14 assisted deliveries (10%). There was a significant correlation of presentation of the second twin (breech or transverse) with cesarean section outcome (*P* = 0.01). In a total of 108 surgical deliveries of the first twin, there were four emergency cesarean sections (3.7%), and in 134 surgical deliveries of the second twin, there were 20 emergency cesareans (14.9%) with 16 (61.5%) being performed in the combined group.

**Table 2:** Indications for cesarean section

	*n*	%
Both twins (*n* = 108)		
Fetal distress	48	44.5
First stage arrest	59	54.6
Placental abruption	1	0.9
Combined delivery (*n* = 26)		
Fetal distress	20	76.9
Second stage arrest	4	15.5
Placental abruption	1	3.8
Cord prolapse	1	3.8

The neonatal characteristics and outcomes for the first and second twins, respectively, are shown in Table 3. The mean gestational age was 258 days (± 12.7). Values for the fetal outcome parameters (pH, base excess, and Apgar scores) were significantly lower in the second twins (*P* < 0.01), but with no additional admissions to neonatal care units. Severe acidosis was rare, with four first twins and seven second twins having an umbilical artery pH below 7.10. The lowest umbilical artery pH was 7.05 in the first twins and 6.78 in the second twin group, due to fetal bradycardia. There was only one first twin with a 5-min Apgar score below 7, but this was observed in seven second twins.

**Table 3:** Neonatal characteristics and outcome for both twins

	First twin	Second twin	*P* value
Birth weight (g)			
Mean	2578.0	2514.9	< 0.01
SD	404.7	452.5	
pH UA			
Mean	7.30	7.25	< 0.01
SD	0.07	0.08	
pH UV			
Mean	7.35	7.30	< 0.01
SD	0.06	0.08	
Base excess			
Mean	− 4.08	− 4.69	< 0.01
SD	2.55	2.89	
Apgar 1 min			
Median	9	9	< 0.01
Range	2–10	1–10	
Apgar 5 min			
Median	10	10	< 0.01
Range	6–10	4–10	
Apgar 10 min			
Median	10	10	< 0.01
Range	7–10	4–10	
Apgar 5 min < 4			
* n*	0	0	
%	0.0	0.0	
Apgar 5 min < 7			
* n*	1	7	
%	0.4	2.8	
Admission to NICU/NIMCU/NEO			
* n*	144	151	0.19
%	58.1	61.1	
Congenital anomaly			
* n*	32	28	0.61
%	12.9	11.3	

*NEO* neonatal ward, *NICU* neonatal intensive care unit, *NIMCU* neonatal intermediate care unit, *pH UA* arterial umbilical cord pH, *pH UV* venous umbilical cord pH, *SD* standard deviation

**Fig. 1:**
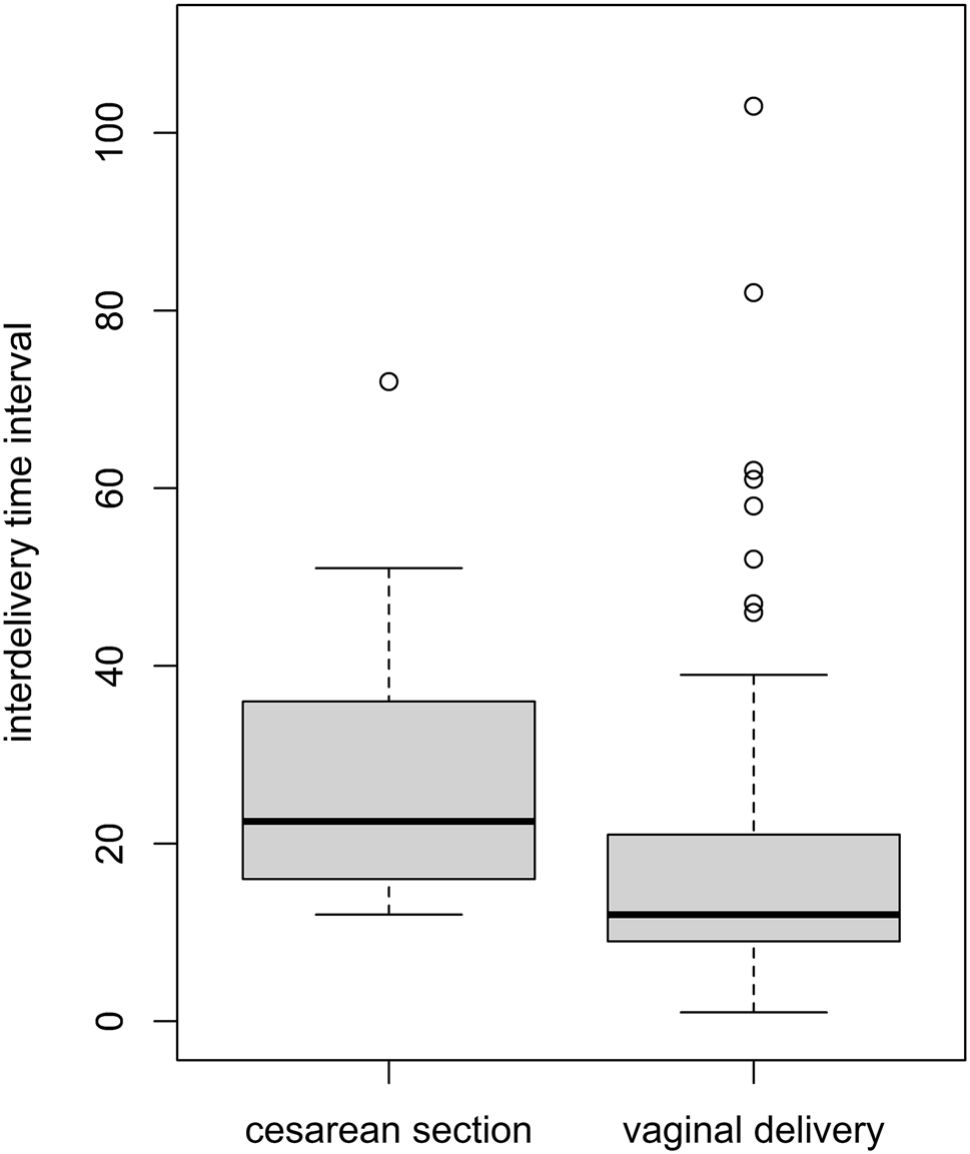
Correlation of interdelivery time interval and birth mode of the second twin. Box plots showing the interdelivery time interval (in min) for second twins born via vaginal or cesarean section delivery (*P* < 0.01). Boxes: interquartile range; horizontal line: median (Q2); whiskers: range; circles: outliers

The mean interdelivery time interval was 11.5 min (± 15.05), and the longest interval was 103 min. In the subgroup with the first twin born via vaginal delivery, the mean interdelivery time interval in the second twin vaginal group was 17.6 min (± 15.79), while in the second twin cesarean section group it was 27.5 min (± 14.36), which is significantly longer (*P* < 0.01). The correlation of interdelivery time interval and birth mode of the second twin is represented in Fig. 1. In the subgroup with the first twin born vaginally, the correlation analysis between interdelivery time intervals and Apgar scores for the second twin did not show any significant correlation between the time interval and differences in the 1-, 5- and 10-min Apgar scores (*ρ* = 0.056, *P* = 0.51; *ρ* = 0.003, *P* = 0.98; *ρ* = 0.090, *P* = 0.29) (Table 4).

**Table 4:** Correlation between the interdelivery time interval and the neonatal outcome for the second twin

	Interdelivery time interval (1st minus 2nd twin; min)		
	1-min Apgar difference	5-min Apgar difference	10-min Apgar difference
Spearman’s correlation coefficient (*ρ*)	0.056	0.003	0.090
*P* value	0.51	0.98	0.29

The subgroup analyses of first and second twins delivered via vaginal delivery in comparison with those with unplanned cesarean deliveries are shown in Table 5. In all, 140 of 248 first twins (56.5%) were born vaginally, and unplanned cesarean sections had to be performed in 108 (43.5%). Neonatal outcome parameters (pH and Apgar scores) were significantly lower in the cesarean section group (*P* < 0.05), with significantly more admissions to neonatal wards in this group. The base excess (BE) was significantly lower in the vaginal group (*P* < 0.05). There was only one first twin with a 5-min Apgar score below 7 in the cesarean section group. The lowest umbilical artery pH (7.05) was observed in the vaginal group. There were significantly more congenital anomalies in the cesarean section group (*P* < 0.05).

**Table 5:** Neonatal characteristics and outcomes for first and second twins (vaginal vs. secondary cesarean)

	First twin			Second twin		
	Vaginal	Secondary cesarean	*P* value	Vaginal	Secondary cesarean	*P* value
Birth weight (g)						
Mean	2681.2	2444.3	< 0.01	2622.1	2423.7	< 0.01
SD	408.3	359.8		440.6	444.0	
pH UA						
Mean	7.30	7.29	< 0.05	7.24	7.26	< 0.05
SD	0.07	0.05		0.09	0.08	
pH UV						
Mean	7.36	7.33	< 0.01	7.29	7.31	0.23
SD	0.06	0.05		0.08	0.08	
Base excess						
Mean	− 4.42	− 3.65	< 0.05	− 5.23	− 4.23	< 0.01
SD	2.61	2.40		2.91	2.81	
Apgar 1 min						
Median	9	9	< 0.01	9	9	0.13
Range	6–10	2–10		4–9	1–10	
Apgar 5 min						
Median	10	10	< 0.01	10	10	< 0.05
Range	7–10	6–10		5–10	4–10	
Apgar 10 min						
Median	10	10	< 0.05	10	10	< 0.05
Range	9–10	7–10		7–10	4–10	
Apgar 5 min < 4						
*n*	0	0		0	0	
%	0.0	0.0		0.0	0.0	
Apgar 5 min < 7						
*n*	0	1		1	6	
%	0.0	0.9		0.9	4.5	
Admission to NICU/NIMCU/NEO						
*n*	65	79	< 0.01	54	97	< 0.01
%	46.4	73.1		47.8	72.4	
Congenital anomaly						
*n*	12	20	< 0.05	6	22	< 0.01
%	8.6	18.5		5.3	16.4	

*NEO* neonatal ward, *NICU* neonatal intensive care unit, *NIMCU* neonatal intermediate care unit, *pH UA* arterial umbilical cord pH, *pH UV* venous umbilical cord pH, *SD* standard deviation

In all, 114 of the 248 second twins (46.0%) were born vaginally (Table 4); unplanned cesarean sections were performed in 134 (54.0%), with 26 combined deliveries (10.5%). Values for neonatal outcome parameters (pH and BE) were lower in the vaginal group, with significant differences in umbilical artery pH (*P* < 0.05) and BE (*P* < 0.01). All of the Apgar scores were lower in the cesarean section group, with significant differences in the 5- and 10-min Apgar scores (*P* < 0.05). There were significantly more admissions to neonatal units in the cesarean group (*P* < 0.01). There were six second twins (4.5%) with a 5-min Apgar score below 7, in comparison with only one (0.9%) in the vaginal group. The lowest umbilical artery pH was 6.78 in the cesarean section group, caused by fetal bradycardia. Significantly more congenital anomalies were noted in the cesarean section group (*P* < 0.01).

## Discussion

The findings of this study confirm the previous evidence that in attempted vaginal deliveries of dichorionic twin pregnancies, the second twin appears to be at greater risk for a poorer neonatal outcome [10, 15, 17, 22, 23]. A study period of ten years was chosen so that there were approximately 200 first and second twins included in the studies, respectively. The standards of the management of twin pregnancies did not change over the study period so a bias seems to be negligible. However, although the results indicate significant differences between the outcome parameters for the first and second twins, the clinical relevance of this needs to be considered. When the mean umbilical artery pH values in the two groups are compared, the significantly lower pH in the second twin group was 7.25, so the differences in pH are small. The lowest umbilical artery pH was 6.78 in the second twin group, due to fetal bradycardia, and there were no other pH findings below 7.0. Analyzing the Apgar scores for both twins does show a significant difference in relation to the ranges. However, the median is exactly the same as in the group of first twins. It is worth mentioning that the Apgar score for postpartum fetal assessment has severe limitations in all preterm births, as it was created for assessing term deliveries. There were only a few twins that met the definitions for severe neonatal asphyxia and low 5-min Apgar scores in the whole patient group included in this study, but this was not only because of the twin birth itself, but also due to intrapartum complications, such as a pathological heart rate or placental abruption, which are never totally preventable. In addition, the number of twins requiring admission to neonatal units was not significantly higher in the second twin group, indicating that the clinical outcome for both twins was comparable. However, newborns in the present study might be transferred either to the neonatal intensive care unit (NICU) or neonatal intermediate care unit (NIMCU), and additionally, some of the twins might also be admitted to a basic neonatal ward (NEO) without intensive care due to the fact of prematurity or maternal gestational diabetes, and not only because of the outcome of delivery.

With regard to interdelivery time intervals, the results of previous studies are contradictory. Some authors have reported a significant correlation between time intervals and the outcome for the second twin, not always depending on monochorionicity [5, 16, 24, 25]. In the present study, there was no significant correlation between longer interdelivery time intervals and lower 1-min, 5-min, and 10-min Apgar scores. Once again, therefore, the clinical relevance and outcome need to be taken into account. Although there is a lack of clear evidence, an upper time limit might be justified. In our hospital, we prefer nonactive management of the second twin, depending on risk factors, such as parity, estimated birth weight, and of course the evaluation of the obstetrician on duty.

Comparison of the outcomes for second twins delivered vaginally with outcomes for those delivered via cesarean section shows similar results. With regard to the 5- and 10-min Apgar scores, there was a significantly better outcome in the vaginal group, but again this did not have any clinical relevance, as the median was the same in the two groups. Interestingly, the umbilical artery pH was significantly higher in the cesarean group. This might be due to the fact that, among 134 unplanned surgical deliveries of the second twin, 108 cesarean sections were conducted on both twins and only 26 involved a combined vaginal delivery of the first twin and cesarean delivery of the second. The indication for cesarean delivery in these 108 cases might therefore have included fetal distress only in the first twin, maternal indications, or unsuccessful vaginal delivery of the first twin—all factors that do not influence the outcome for the second twin. Notably, the analysis showed that 20 out of 24 emergency cesarean sections were on the second twin, with 16 of these being conducted in the combined vaginal–surgical delivery group. It might therefore be concluded that the second twin is at high risk of being delivered via emergency cesarean section. This is because cesarean section in a combined delivery mode is often carried out due to emergency conditions, such as a pathological fetal heart rate, umbilical cord prolapse, or placental abruption [26]. All in all, 1.6% of the first twins and 8.1% of the second twins have been delivered via emergency cesarean section. In comparison, in singleton pregnancies a rate of emergency cesarean section of 1.4% is registered in our study period.

With regard to the presentation of the second twin, transverse or breech presentation is also challenging. In the group of second twins, there were 23 with transverse presentation and 54 with breech presentation. 17 of the second twins were born via vaginal breech delivery. We agree with Bogner et al. that a noncephalic presentation of the second twin does not in itself significantly influence the clinical outcome [18]. Following a decrease in singleton breech deliveries as a consequence of the Term Breech Trial, the number of obstetricians who offer vaginal breech delivery in singletons and twins is declining [27]. Bogner et al. concluded that the presentation of the second twin does not significantly influence its obstetric outcome. However, serious adverse outcomes for the second twin were rare in the study [18]. In our department, similarly, the number of obstetricians offering vaginal breech delivery has been decreasing rapidly in recent years. This is due to the increasing numbers of external cephalic versions and decreasing numbers of vaginal breech deliveries, so that there are few opportunities to train young obstetricians in breech delivery skills.

Subgroup analysis of vaginal versus surgical delivery of the first twin shows that there is an increased risk of a poorer outcome if there is an intrapartum change of birth mode from attempted vaginal delivery to secondary cesarean section. Although the values for the outcome parameters are significantly lower in the cesarean section group, again there were only small differences in the pH and Apgar scores, weakening the clinical relevance of the finding. In addition, the significantly higher rates of admission to neonatal units in the surgical group may be explained by the significantly larger numbers of congenital anomalies reported.

The present study has inherent limitations associated with its retrospective design. As the results diverge from those of previous studies, there is a clear need for further prospective studies on the topic including larger numbers of patients. Another limitation that needs to be considered is that the study included a very heterogeneous group of neonates, with fetal anomalies not being excluded. However, the malformations reported in the group were mild, such as limb anomalies or small ventricular septal defects; there were no severe anomalies or severe congenital heart defects such as tetralogy of Fallot, for example. In the study collective, we wanted to include all twin pregnancies with an attempt of a vaginal birth. No severe anomalies have been registered as in those cases, a primary cesarean section is normally performed, so there should be no bias in the analysis. In addition, even in a tertiary center, the clinicians treating the women often have various levels of obstetric skills, so that their experience and evaluation of the intrapartum situation could potentially have influenced their decision making. One strength of the present study is the large number of attempted vaginal deliveries included, and an innovative aspect of it is the fact that the subgroup analysis does not include comparison with a group receiving planned cesarean sections. This makes it easier to investigate whether women and their neonates experience any disadvantages if the birth mode needs to be changed due to the course of delivery.

## Conclusion

Although there appears to be a significant risk for a poorer outcome when there are intrapartum changes of birth mode, especially for second twins in attempted vaginal deliveries, this study shows that the clinical relevance of these findings is negligible. In general, serious neonatal morbidity is rare in dichorionic twin pregnancies. Spontaneous delivery can therefore be recommended in women with dichorionic pregnancies. Providing the patients with detailed information about potential intrapartum complications that may require emergency measures is therefore mandatory. Limiting the interdelivery time interval is not obligatory, but may be justified. Appropriate training for young obstetricians is advisable in any case.

## Data Availability

The datasets used and/or analyzed during the current study are available from the corresponding author on reasonable request.

## Abbreviations

BE: Base excess
BMI: Body mass index
NEO: Neonatal ward
NICU: Neonatal intensive care unit
NIMCU: Neonatal intermediate care unit
pH UA: Arterial umbilical cord pH
pH UV: Venous umbilical cord pH
SD: Standard deviation
